# Thymol targeting interleukin 4 induced 1 expression reshapes the immune microenvironment to sensitize the immunotherapy in lung adenocarcinoma

**DOI:** 10.1002/mco2.355

**Published:** 2023-08-30

**Authors:** Tong Li, Jie Shi, Longlong Wang, Xuan Qin, Rui Zhou, Ming Dong, Fan Ren, Xin Li, Zihe Zhang, Yanan Chen, Yanhua Liu, Yongjun Piao, Yi Shi, Song Xu, Jun Chen, Jia Li

**Affiliations:** ^1^ Department of Lung Cancer Surgery Tianjin Medical University General Hospital Tianjin China; ^2^ Tianjin Key Laboratory of Lung Cancer Metastasis and Tumor Microenvironment Lung Cancer Institute Tianjin Medical University General Hospital Tianjin China; ^3^ School of Medicine Nankai University Tianjin China; ^4^ Department of Thyroid and Neck Tumor Tianjin Medical University Cancer Institute and Hospital Tianjin China

**Keywords:** IL4I1, immune microenvironment, lung adenocarcinoma, PD‐1 blockade, thymol

## Abstract

Immune checkpoint blockades are the most promising therapy in lung adenocarcinoma (LUAD). However, the response rate remains limited, underscoring the urgent need for effective sensitizers. Interleukin 4 induced 1 (IL4I1) is reported to have immunoinhibitory and tumor‐promoting effects in several cancers. However, the targetable value of IL4I1 in sensitizing the immunotherapy is not clear, and there is a lack of effective small molecules that specifically target IL4I1. Here, we show that silencing IL4I1 significantly remodels the immune microenvironment via inhibiting aryl hydrocarbon receptor (AHR) signaling, thereby enhancing the efficacy of anti‐PD‐1 antibody in LUAD, which suggests that IL4I1 is a potential drug target for the combination immunotherapy. We then identify thymol as the first small molecule targeting IL4I1 transcription through a drug screening. Thymol inhibits the IL4I1 expression and blocks AHR signaling in LUAD cells. Thymol treatment restores the antitumor immune response and suppresses the progression of LUAD in an orthotopic mouse model. Strikingly, the combination treatment of thymol with anti‐PD‐1 antibody shows significant tumor regression in LUAD mice. Thus, we demonstrate that thymol is an effective small molecule to sensitize the PD‐1 blockade in LUAD via targeting IL4I1, which provides a novel strategy for the immunotherapy of LUAD.

## Graphical abstract

IL4I1 is a promising drug target for the combination immunotherapy in lung adenocarcinoma (LUAD). Thymol is identified as an effective natural small molecular that can significantly inhibit the transcription of IL4I1. Thymol treatment dramatically restores the antitumor immune response to sensitize the PD‐1 blockade in LUAD via targeting IL4I1‐mediated aryl hydrocarbon receptor (AHR) activation, which provides a novel strategy for the combination immunotherapy for LUAD.

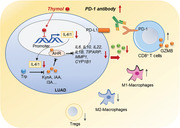

## 1 INTRODUCTION

Non‐small cell lung cancer (NSCLC) has the highest mortality among cancers worldwide, and the most common pathological subtype of NSCLC is lung adenocarcinoma (LUAD).^1^ While immune checkpoint blockades (ICBs), particularly anti‐PD‐1/PD‐L1 antibodies, have emerged as the most promising antitumor therapy, the response rate in NSCLC patients remains relatively low, hovering around 20%.^2^ Consequently, there exists a critical need to develop effective sensitizers to enhance the efficacy of combination immunotherapy for LUAD.

The infiltration and cytotoxicity of CD8^+^ T cells in the tumor microenvironment (TME) define the immune status (i.e., inflamed, immune exclude, or immune desert) of the tumor and can promote or hinder the response to ICBs.^3^ Besides, the dysregulation of other antitumor immune cells, such as M1‐like macrophages and natural killer (NK) cells, and the increased infiltration of immunosuppressive cells, such as M2‐like macrophages and regulatory T cells (Tregs), in TME also contribute to the resistance of ICBs.^3^, ^4^ Accumulating evidence strongly suggests that TME significantly affects the clinical efficacy of immunotherapy. Therefore, remodeling the TME to switch the immune exclude or immune desert tumor to an inflamed tumor is an effective strategy to sensitize the ICB therapy.^5^

The tryptophan (Trp)/aryl hydrocarbon receptor (AHR) pathway has been identified as a key driver of immunosuppressive microenvironment in various cancer types. Metabolites derived from Trp act as endogenous ligands that bind to AHR, a cellular sensor. Upon ligand binding, AHR translocates into the nucleus and regulates target gene transcription, which is critical for biological processes, including normal development, immunity, and cancer development.^6^, ^7^, ^8^, ^9^ The Trp/AHR pathway links the abnormal metabolism of tumor cells to the immunosuppressive TME, and has been a promising target for drug development in cancer immunotherapy.^10^, ^11^ Currently, there are several AHR inhibitors that have been developed. However, out of these inhibitors, only three, namely, IK‐175, BAY2416964, and SR‐1, are in clinical trials for the treatment of cancers and for expanding hematopoietic stem cells currently.^12^ The complex regulatory roles of AHR in normal physiological processes have led researchers to consider the Trp catabolic enzymes as more attractive targets for cancer therapy, rather than directly targeting AHR. Indoleamine 2,3‐dioxygenase 1 (IDO1), IDO2, and tryptophan 2,3‐dioxygenase are the main Trp catabolic enzymes. IDO1, which is highly expressed in tumors and associated with resistance to ICBs, has been considered as a promising target for cancer therapy.^13^, ^14^ However, increasing evidence shows that cancer patients cannot benefit from the treatment of IDO1 inhibitors alone or in combination with ICBs.^15^

A recent study has pointed out that instead of IDO1, interleukin 4 induced 1 (IL4I1) is the novel and the main Trp‐catabolic enzyme in glioma and chronic lymphocytic leukemia, which explains the major reason why the clinical trials of IDO1 inhibitors are a failure.^16^ Previous studies have also shown that IL4I1 is a crucial player in the immunosuppressive TME, which promotes the progression of breast cancer, ovarian cancer, melanoma, and colon cancer.^17^, ^18^, ^19^, ^20^, ^21^, ^22^ IL4I1 acts as the main regulator of Trp/AHR signaling and promotes the immune evasion of several cancer types, underscoring its significance as a key drug target.

However, the role of IL4I1 in LUAD is still keep largely unknown. Despite being recognized as a key immunoinhibitory enzyme in various cancers, the targetable value of IL4I1 in sensitizing the immunotherapy is still not clear. Additionally, there currently are no effective small molecules targeting IL4I1. In the present study, we have demonstrated the significance of IL4I1 as a promising drug target for the combination immunotherapy in LUAD. Moreover, we have made a significant breakthrough by identifying thymol as the first small molecule targeting IL4I1 transcription through a drug screening. Thymol treatment dramatically sensitizes the PD‐1 blockade in LUAD by targeting IL4I1‐mediated AHR activation, which provides a novel strategy for the combination immunotherapy in LUAD.

## 2 RESULTS

### 2.1 IL4I1 is a promising drug target to sensitize the ICBs in LUAD

To evaluate the role of IL4I1 in LUAD patients, we collected the primary LUAD tissues and paired adjacent normal tissues (NTs) from LUAD patients. Quantitative real‐time PCR (qRT‐PCR), western blot, and immunohistochemistry (IHC) staining show that the expression of IL4I1 is dramatically upregulated in primary tumors (Figure S1A–C). The data from the GTEx and TCGA databases also show the elevated expression of IL4I1 in LUAD tissues (Figure S1D,E). The protein levels of IL4I1 are increased with the grade of LUAD (Figure S1F). In addition, IL4I1 is significantly negatively correlated with the overall survival and progression‐free survival (PFS) of LUAD patients (Figure S1G,H).

**FIGURE 1:**
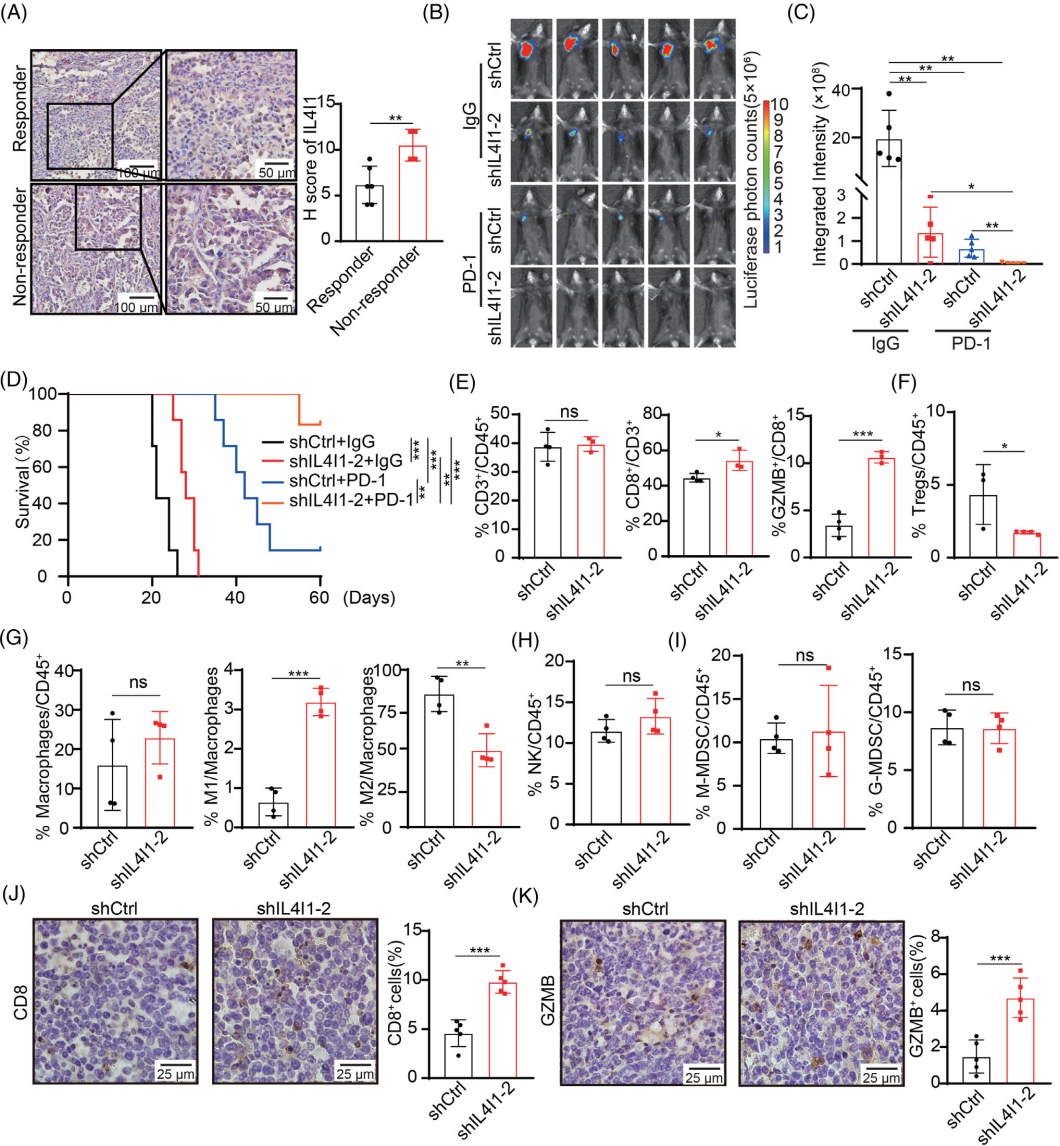
IL4I1 modulates the efficacy of anti‐PD‐1 antibody via modulating the immune microenvironment in LUAD. (A) Representative images of IHC analysis of IL4I1 in tumors of LUAD patients who responded to anti‐PD‐1/PD‐L1 treatment (responder, *n* = 6) and those who did not (nonresponder, *n* = 4), and quantification by H‐score. (B, C) shCtrl and shIL4I1‐2 LLC‐luciferase cells were orthotopically injected in the right lung of C57BL/6 mice. Ten milligram per kilogram anti‐PD‐1 antibody or IgG was intraperitoneally injected every 1 week from day 5 after tumor injection. Bioluminescence images of mice at day 21 after tumor injection are shown in (B) and the statistical results of average integrated bioluminescence intensity are shown in (C) (*n* = 5). (D) The survival curves of C57BL/6 mice (*n* = 7). (E) The quantification of CD3^+^ T cells, CD8^+^ T cells, and granzyme B^+^ (GZMB^+^) T cells in LUAD tumors formed in C57BL/6 mice. (F–I) The quantification of regulatory T cells (Tregs, F), macrophages and M1‐like, M2‐like subpopulations (G), natural killer (NK) cells (H), monocytic‐myeloid‐derived suppressor cells (M‐MDSCs) and granulocytic‐MDSC (G‐MDSC, I) in LUAD tumors formed in C57BL/6 mice. (J, K). Representative images of IHC analysis of CD8 (J) and GZMB (K) in LUAD tumors formed in C57BL/6 mice with quantification analyses. Quantification data are plotted as means ± SEM. ns, not significant; *p < 0.05; **p < 0.01; ***p < 0.001, by unpaired two‐sided Student's *t*‐test for A, C, E–K, by log‐rank test for D.

To further evaluate the association between IL4I1 expression and the efficacy of ICBs, we collected 10 pretreated tumors from LUAD patients who responded to anti‐PD‐1/PD‐L1 treatment (responder, *n* = 6) and those who did not (nonresponder, *n* = 4). The IHC staining shows that the expression of IL4I1 is much higher in tumors from nonresponders compared with those from responders, suggesting that IL4I1 expression is negatively correlated with the efficacy of ICBs in LUAD patients (Figure 1A).

We further knock down IL4I1 in Lewis lung cancer (LLC) cells to investigate the role of IL4I1 in sensitizing the PD‐1 blockade in an orthotopic LUAD murine model (Figure S2A,B). Strikingly, the tumors are almost completely repressed in mice injected with IL4I1‐silenced (shIL4I1‐2) LLC cells upon the treatment of anti‐PD‐1 antibody (Figure 1B,C). Besides, the anti‐PD‐1 antibody greatly enhances the survival of mice injected with IL4I1‐silenced LLC cells (Figure 1D).

Since very little IL4I1‐silenced LLC tumor remained in mice treated with anti‐PD‐1 antibody, we measure immune cell infiltration in tumors from untreated mice. The fluorescence activating cell sorter (FACS) analyses show that silencing IL4I1 in LLC cells significantly increases the proportion of CD8^+^ T cells, granzyme B^+^ (GZMB^+^) CD8^+^T cells, and M1‐like macrophages, while decreases the proportion of Tregs and M2‐like macrophages (Figure 1E–G and Figure S3A–C). The ratios of NK cells, monocytic‐myeloid‐derived suppressor cells (M‐MDSCs), and granulocytic‐MDSC (G‐MDSC) have no significant change upon the silenced of IL4I1 (Figure 1H,I and Figure S3D,E). The IHC staining confirms more CD8^+^ cells and GZMB^+^ cells infiltration in IL4I1‐silenced LUAD tumors (Figure 1J,K). We further detect the proportion of CD8^+^ T cells in spleen, and find that the ratio of CD8^+^/CD3^+^ T cells is increased in the spleen of mice injected with IL4I1‐silenced LLC cells with PD‐1 blockade (Figure S4A). We also examine the relevance of IL4I1 with the CD8^+^ T cells infiltration in human LUAD clinical specimens by IHC staining and confirm the increased CD8^+^ T cells infiltration in LUAD tumors with lower IL4I1 expression (Figure S5A).

Taken together, these data suggest that silencing IL4I1 significantly reactivates the immune microenvironment, thereby improving the efficacy of PD‐1 blockade in LUAD.

### 2.2 IL4I1 is closely associated with the AHR activity in LUAD

**FIGURE 2:**
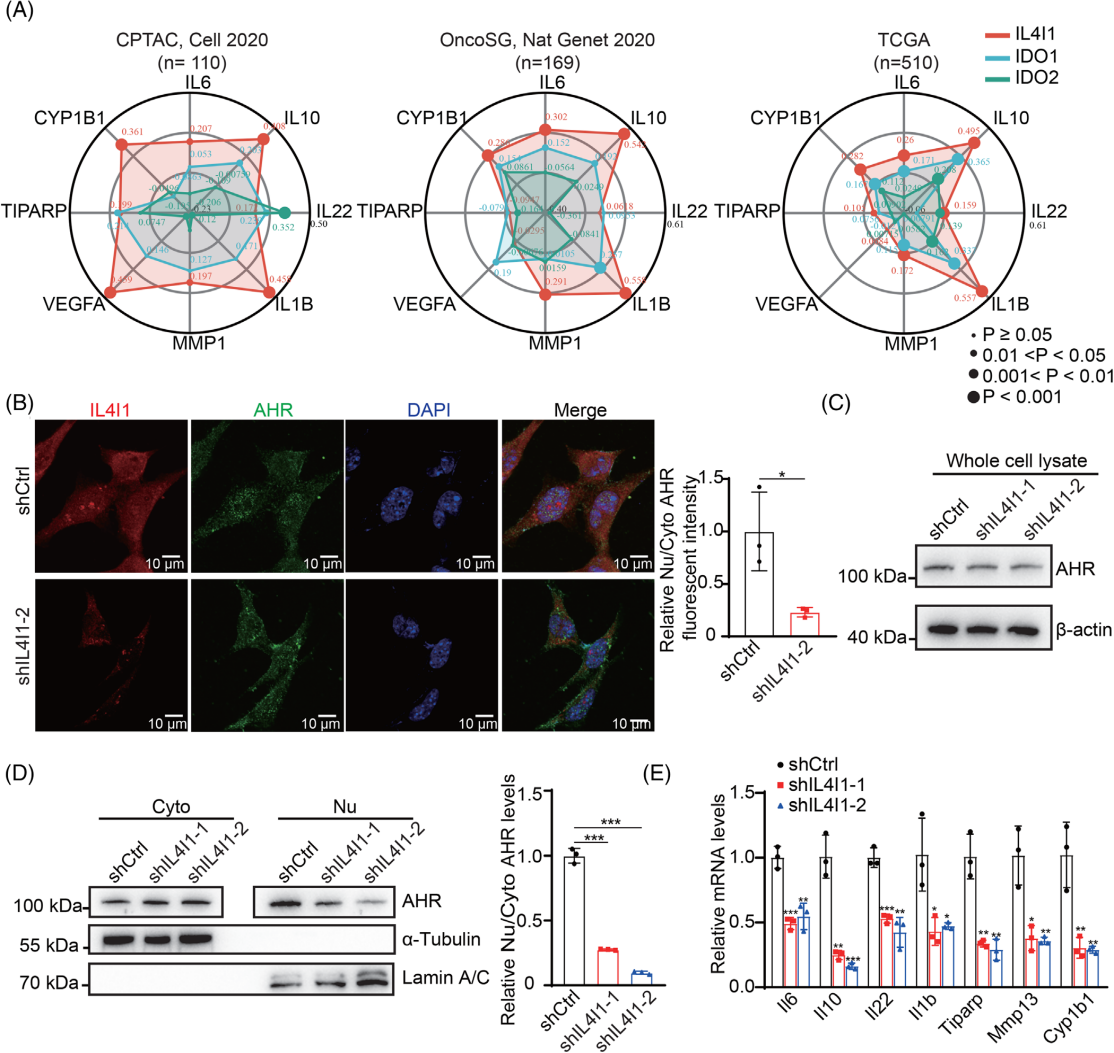
IL4I1 is closely associated with the AHR activity in LUAD. (A) The Spearman's correlation analyses of the *IL4I1*, *IDO1*, and *IDO2* expression with AHR target genes in LUAD patients from CPTAC (*n* = 110), OncoSG (*n* = 169), and TCGA databases (*n* = 510). (B) IF staining of IL4I1 (red), AHR (green), and DAPI (blue) in shCtrl and shIL4I1‐2 LLC cells, and the quantification results of the fluorescent intensity of AHR in the nucleus (nu)/cytoplasm (cyto). (C) The protein levels of total AHR in shCtrl and shIL4I1 LLC cells. (D) The protein levels of AHR in cytoplasm (cyto) and nucleus (nu) in shCtrl and shIL4I1 LLC cells, and the quantification results of the relative nu/cyto AHR levels. (E) The mRNA levels of AHR target genes in shCtrl and shIL4I1 LLC cells. Quantification data are plotted as means ± SEM. ns, not significant; *p < 0.05; **p < 0.01; ***p < 0.001, by unpaired two‐sided Student's *t*‐test.

The key enzymes involved in Trp metabolism are context and cancer‐dependent. However, the key enzyme involved in Trp/AHR activation in LUAD is still not clear. We compare the correlations between IL4I1, IDO1, and IDO2 expression and the AHR activity in LUAD. The coexpression analyses from three LUAD databases (CPTAC, *n* = 110; OncoSG, *n* = 169; and TCGA databases, *n* = 510) show that IL4I1 associates more closely with AHR target genes (*IL6*, *IL10*, *IL22*, *IL1B*, *TIPARP*, *MMP1*, *CYP1B1*, and *VEGFA*) than IDO1 or IDO2 (Figure 2A). Moreover, the western blot analyses and the immunofluorescence (IF) staining show that silencing IL4I1 in LLC cells significantly blocks the nuclear translocation of AHR (Figure 2B–D). The mRNA levels of AHR target genes (*Il6*, *Il10*, *Il22*, *Il1b*, *Tiparp*, *Mmp13*, and *Cyp1b1*) are dramatically decreased in IL4I1‐silenced LLC cells (Figure 2E). These data show that IL4I1 is a key modulator for AHR activation in LUAD.

### 2.3 Thymol is identified as an effective small compound targeting IL4I1 expression via a high‐throughput drug screening

**FIGURE 3:**
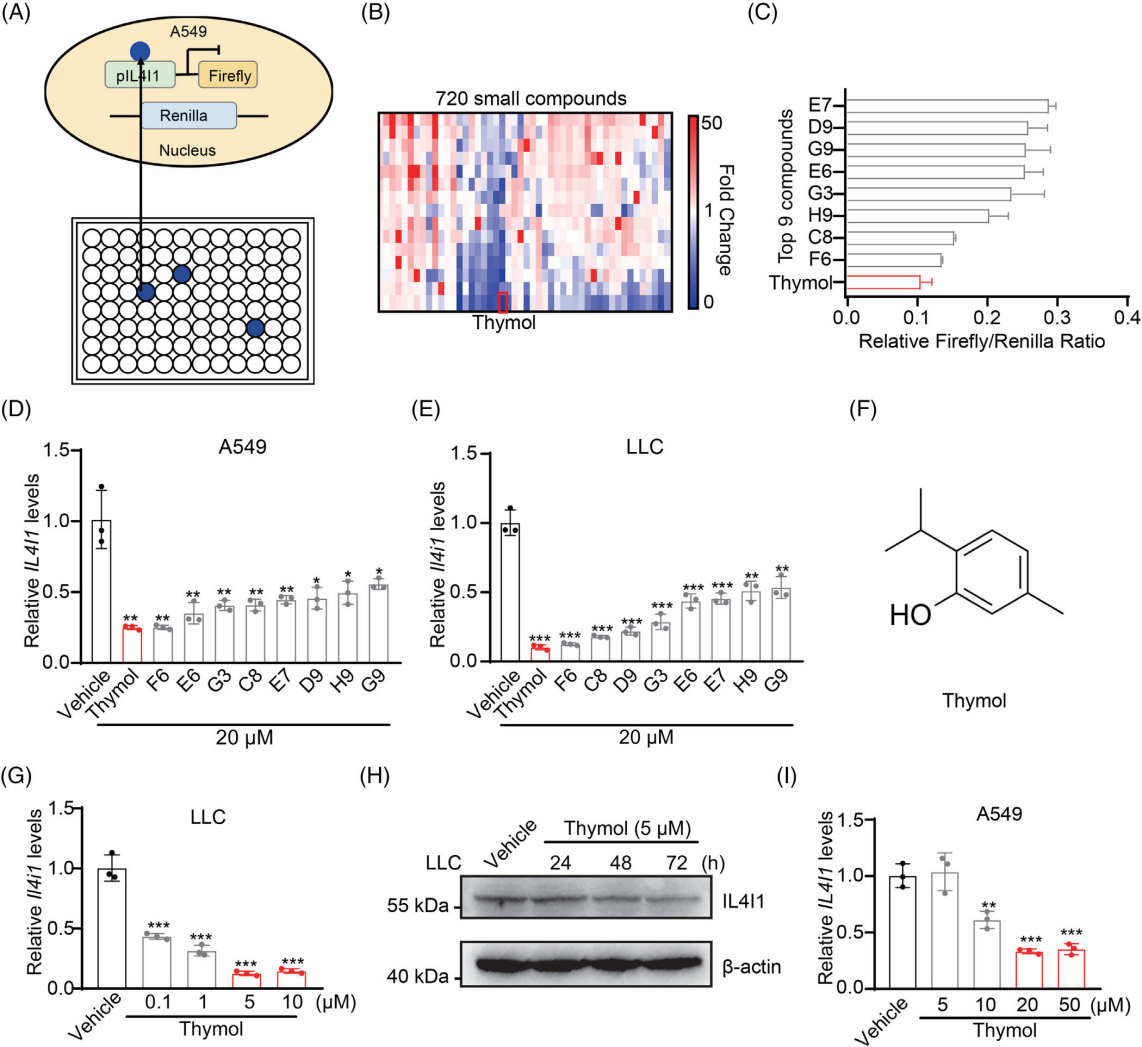
Thymol is an effective small molecular targeting IL4I1 expression. (A) Diagram illustrating system for screening small molecules targeting IL4I1 expression from drug library containing 720 natural small molecules. (B) Heatmap of 720 compounds (20 μM) that regulate IL4I1 expression based on firefly/renilla ratio. (C) The top nine compounds that can inhibit the reporter gene expression to less than 30% were regarded as effective compounds. (D) The mRNA levels of *IL4I1* in A549 cells treated with 20 μM effective compounds for 24 h. (E) The mRNA levels of *Il4i1* in LLC cells treated with 20 μM effective compounds for 24 h. (F) The chemical structure of thymol. (G) The mRNA levels of *Il4i1* in LLC cells treated with thymol for 24 h with different concentrations. (H) The protein levels of IL4I1 in LLC cells treated with 5 μM thymol in different times. (I) The mRNA levels of *IL4I1* in A549 cells treated with thymol for 24 h with different concentrations.

IL4I1 is a hopeful therapeutic target in LUAD, but there is still no effective small molecular targeting IL4I1 expression. To screen effective small compounds targeting IL4I1 expression, human LUAD cells (A549) are used to establish a cell‐based screening system by stably transfected with a firefly luciferase reporter gene driven by IL4I1 promoter region (Figure 3A). We screen the drug library containing 720 natural small molecules using the A549‐based system with a drug concentration of 20 μM. We identify nine compounds that can inhibit the reporter gene expression to less than 30% (Figure 3B,C). Among the nine compounds, thymol shows the highest inhibitory activity to the reporter gene (Figure 3C). We further examine the inhibitory activity of the nine compounds on the endogenous IL4I1 expression in A549 and LLC cells, and find that thymol is still the highest‐ranked hit compound (Figure 3D–F). Thymol inhibits the IL4I1 expression in a dose‐ and time‐dependent manner (Figure 3G–I). The optimum effective concentration of thymol for the inhibition of IL4I1 is 5 μM for LLC cells and 20 μM for A549 cells (Figure 3G,I). We, therefore, identify thymol as the effective compound targeting IL4I1 expression.

### 2.4 Thymol significantly inhibits AHR signaling in LUAD cells

**FIGURE 4:**
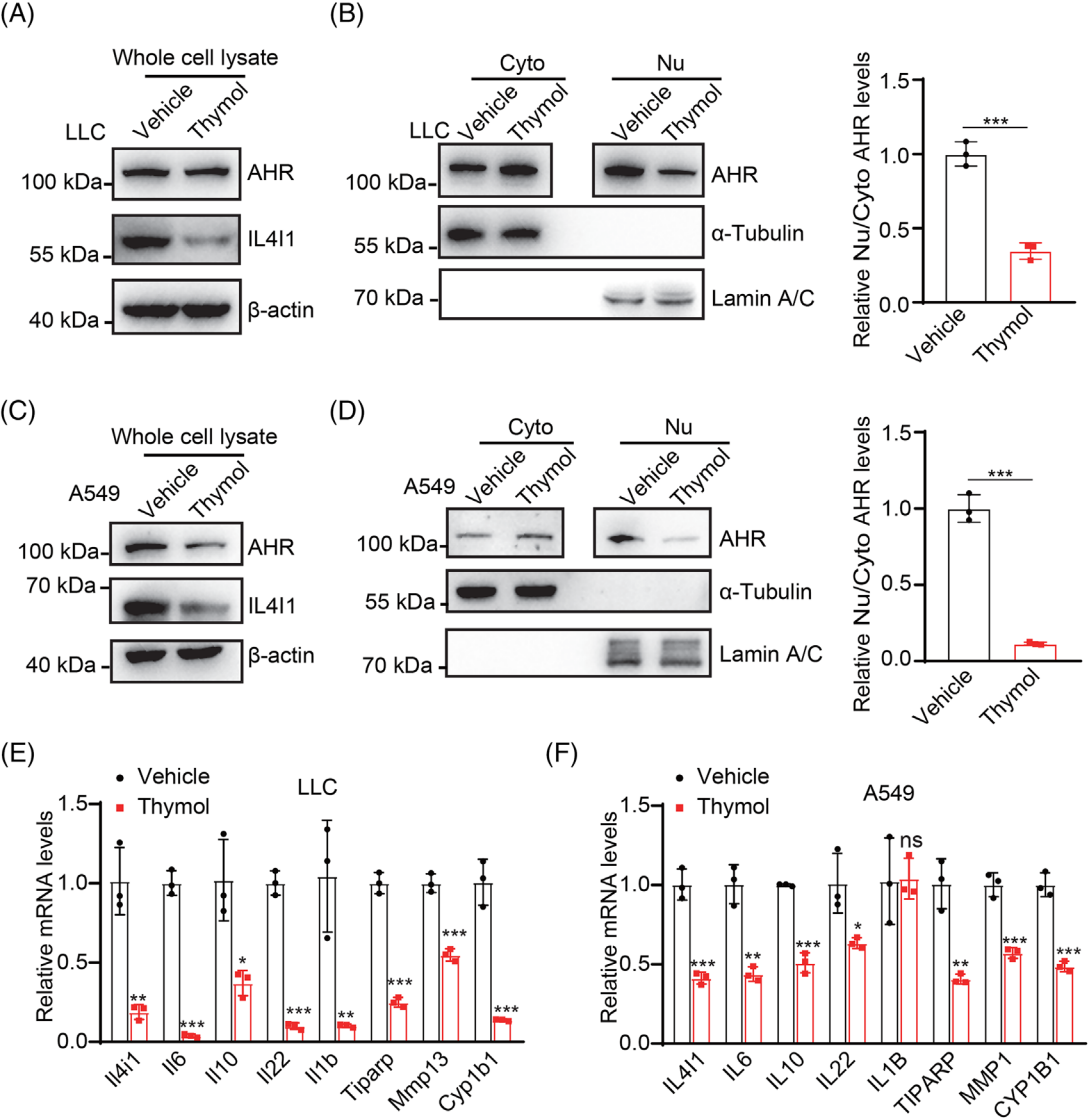
Thymol significantly inhibits AHR signaling in LUAD cells. (A) The protein levels of total AHR in LLC cells treated with 5 μM thymol for 48 h. (B) The protein levels of AHR in nucleus (nu) and cytoplasm (cyto) of LLC cells treated with 5 μM thymol for 48 h, and the quantification results of the relative nu/cyto AHR levels. (C) The protein levels of total AHR in A549 cells treated with 20 μM thymol for 48 h. (D) The protein levels of AHR in nucleus (nu) and cytoplasm (cyto) of A549 cells treated with 20 μM thymol for 48 h, and the quantification results of the relative nu/cyto AHR levels. (E) The mRNA levels of AHR target genes in LLC cells treated with 5 μM thymol for 48 h. (F) The mRNA levels of AHR target genes in A549 cells treated with 20 μM thymol for 48 h. Quantification data are plotted as means ± SEM. ns, not significant; *p < 0.05; **p < 0.01; ***p < 0.001, by unpaired two‐sided Student's *t*‐test.

We further detect the effect of thymol on AHR signaling in vitro. As expected, 5 μM of thymol significantly inhibits the protein levels of IL4I1 and blocks the nuclear translocation of AHR in LLC cells (Figure 4A,B). Consistently, 20 μM of thymol shows a strong effect on the inhibition of IL4I1 protein and AHR signaling in A549 cells (Figure 4C,D). The mRNA levels of AHR target genes in LLC cells and A549 cells are also dramatically decreased upon the treatment of thymol (Figure 4E,F). Therefore, these data suggest that thymol is an effective small compound targeting IL4I1 expression and inhibiting AHR signaling in LUAD cells.

### 2.5 Thymol inhibits the epithelial‐mesenchymal transition and the motility activity in LUAD cells

It has been reported that the activation of AHR signaling is related with the motility ability of cancer cells, so we detect the effect of thymol on the migration of LUAD cells. Five micromoles of thymol significantly inhibits the migration ability of LLC cells (Figure S6A). The expression of epithelial markers is increased, while the expression of mesenchymal markers is decreased in thymol‐treated LLC cells (Figure S6B). Besides, thymol treatment does not affect the proliferation ability of LLC cells (Figure S6C). Consistently, 20 μM of thymol also inhibits the epithelial‐mesenchymal transition and the motility activity in A549 cells without affecting the proliferation ability (Figure S6D–F).

### 2.6 Thymol dramatically sensitizes the PD‐1 blockade in an orthotopic mouse LUAD model

**FIGURE 5:**
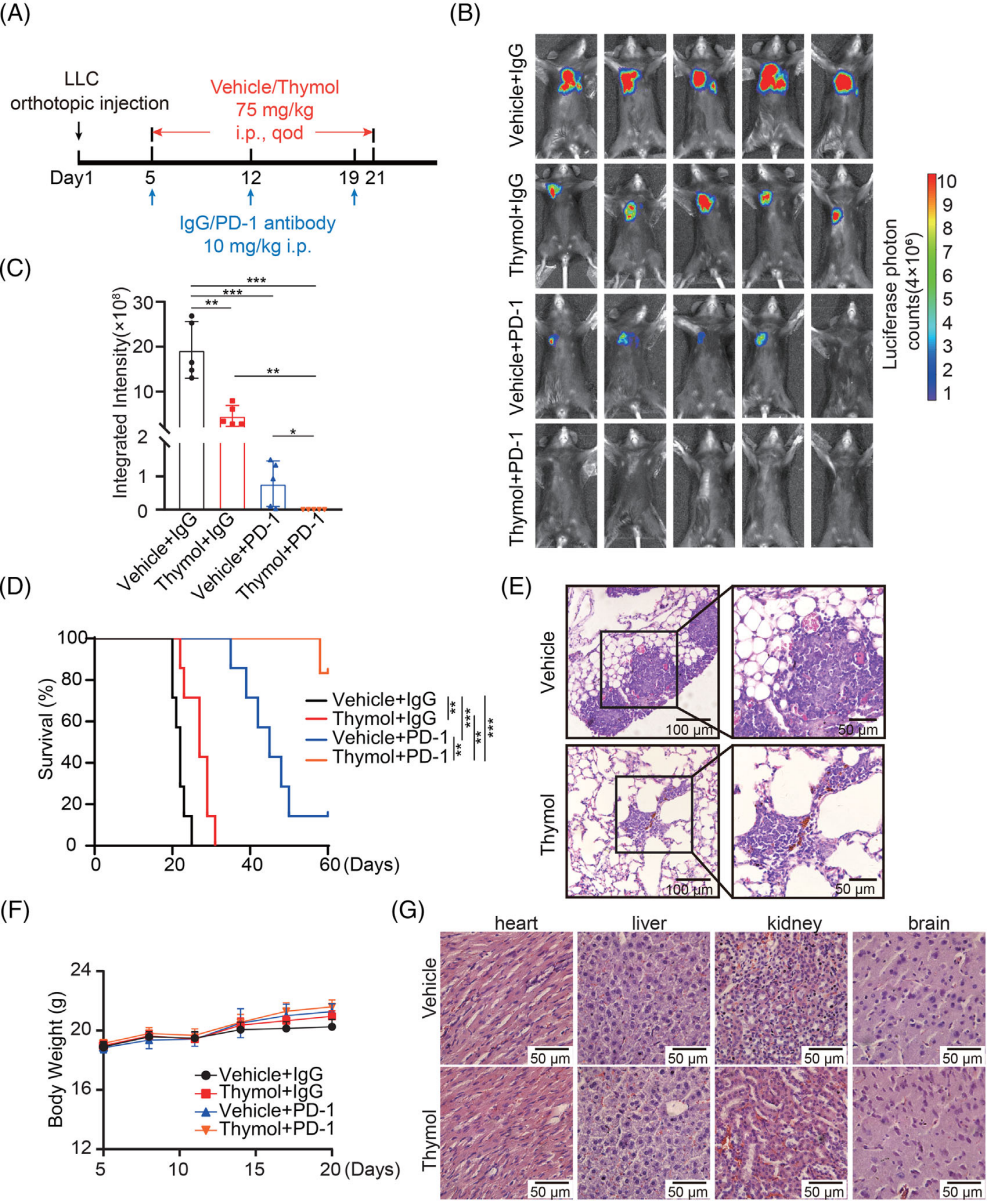
Thymol dramatically improves the efficacy of anti‐PD‐1 antibody. (A) Schematic of the experiments. LLC‐luciferase cells were orthotopically injected in the right lung of C57BL/6 mice. Ten milligram per kilogram anti‐PD‐1 antibody or IgG was intraperitoneally injected every 1 week from day 5 after tumor injection. Seventy‐five milligram per kilogram thymol or control vehicle was intraperitoneally injected every 2 days from day 5 after tumor injection. qod, every other day; i.p., intraperitoneal injection. (B, C) Bioluminescence images of mice at day 21 after tumor injection (B) and the statistical results (C) of average integrated bioluminescence intensity (*n* = 5). (D) The survival curves of C57BL/6 mice (*n* = 7). (E) The H&E staining of the tumors from mice treated with vehicle or thymol. (F) The body weight of mice upon the treatment. (G) The H&E staining of heart, liver, kidney, and brain from mice treated with vehicle or thymol. ns, not significant; *p < 0.05; **p < 0.01; ***p < 0.001, by unpaired two‐sided Student's *t*‐test for C, by log‐rank test for D.

To further detect the role of thymol in sensitizing the PD‐1 blockade in LUAD, we test the efficacy of thymol single (75 mg/kg) or in combination with anti‐PD‐1 antibody in the treatment of C57BL/6 mice orthotopically transplanted with LLC‐luciferase cells (Figure 5A). The bioluminescence analyses show that single thymol treatment can significantly suppress the progression of LUAD (Figure 5B,C). Strikingly, compared with a single treatment with thymol or anti‐PD‐1 antibody, combination treatment dramatically suppresses the progression of LUAD and greatly enhances the survival of mice (Figure 5B–D). The tumors are almost completely repressed upon the combination therapy. The hematoxylin and eosin (H&E) staining is used to assess the histology of the tumors treated with vehicle or thymol (Figure 5E). Besides, no obvious toxicity is observed in mice under the treatment of thymol by monitoring the body weight change, the pathologic changes of major organ, and the serum biochemical indexes (Figure 5F,G and Table S1). Therefore, these data suggest that thymol is an effective small molecule to sensitize the anti‐PD‐1 antibody treatment in LUAD.

### 2.7 Thymol targeting IL4I1 expression significantly remodels the immune microenvironment of LUAD

**FIGURE 6:**
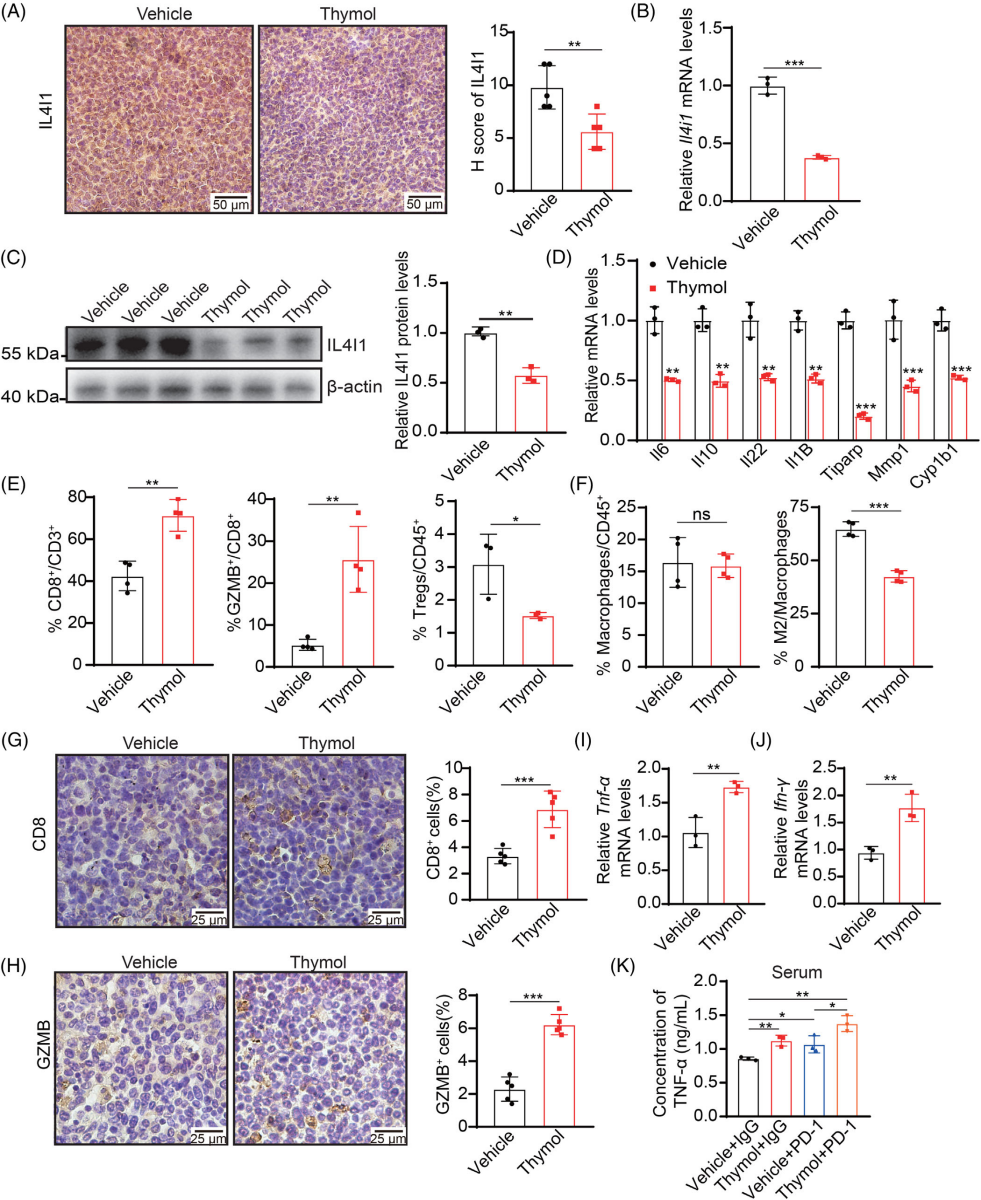
Thymol treatment inhibits the IL4I1 expression and reshapes the immune microenvironment in LUAD tumors. (A) Representative images of IHC analysis of IL4I1 in tumors treated with vehicle or thymol, and quantification by H‐score. (B, C) The mRNA (B) and protein (C) levels of IL4I1 in tumors treated with vehicle or thymol. (D) The mRNA levels of AHR target genes in tumors treated with vehicle or thymol. (E, F) Flow cytometry analyses of CD8^+^ T cells, GZMB^+^ T cells, Tregs, macrophages, and M2‐like subpopulations in tumors treated with vehicle or thymol. (G, H) Representative images and quantification analyses of IHC analyses of CD8 (G) and GZMB (H) in tumors treated with vehicle or thymol. (I, J) The mRNA levels of *Tnf‐α* (I) and *Ifn‐γ* (J) in tumors treated with vehicle or thymol. (K) The concentration of serum TNF‐α upon the treatment. Quantification data are plotted as means ± SEM. ns, not significant; *p < 0.05; **p < 0.01; ***p < 0.001, by unpaired two‐sided Student's *t*‐test.

We further detect the expression of IL4I1 in tumors treated with thymol. IHC staining shows the lower expression of IL4I1 in thymol‐treated tumors (Figure 6A). The mRNA and protein levels of IL4I1 are significantly decreased in tumors upon the treatment of thymol (Figure 6B,C). The mRNA levels of AHR target genes are also significantly decreased in thymol‐treated tumors (Figure 6D).

The FACS analyses of tumor‐infiltrating leukocytes show that thymol treatment significantly increases the proportion of CD8^+^ T cells, especially GZMB^+^ CD8^+^ T cells, and decreases the proportion of Tregs and M2‐like macrophages (Figure 6E,F and Figure S7). The IHC staining confirms more CD8^+^ cells and GZMB^+^ cells infiltration in thymol‐treated tumors (Figure 6G,H). The mRNA levels of inflammatory cytokines, including tumor necrosis factor‐α (*Tnf‐α)* and interferon‐γ (*Ifn‐γ)*, are also increased in tumors upon the treatment of thymol (Figure 6I,J). In addition, the enzyme‐linked immunosorbent assay (ELISA) assay shows that the combined treatment of thymol and PD‐1 significantly boosts the production of serum TNF‐α (Figure 6K). Therefore, these data demonstrate that thymol targeting IL4I1 expression remodels the immune microenvironment to sensitize the PD‐1 blockade in LUAD.

**FIGURE 7:**
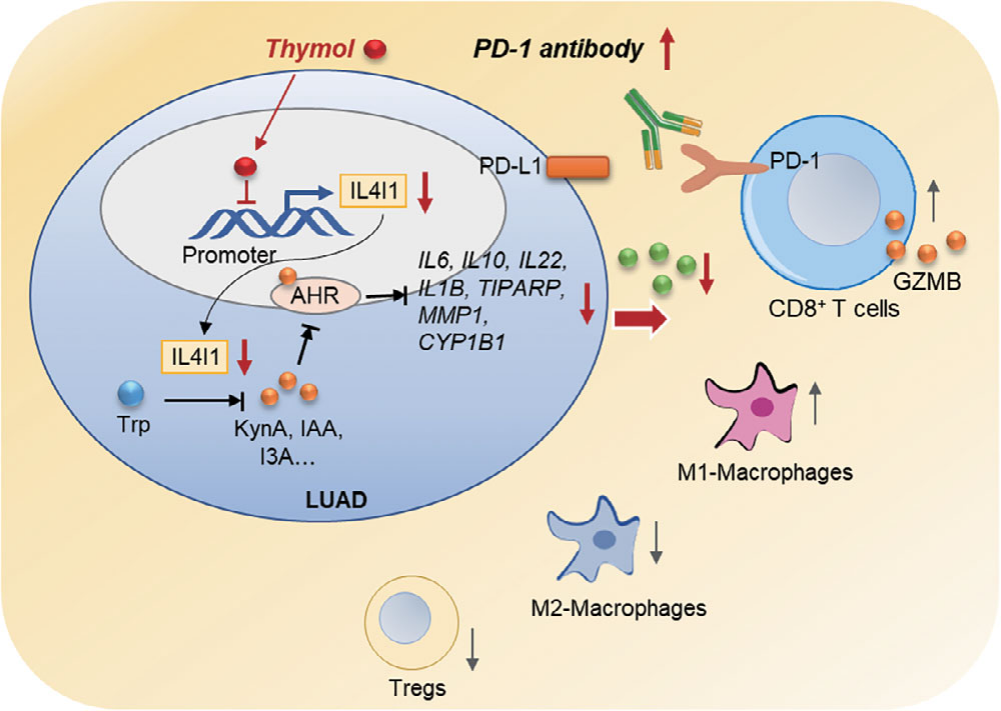
Schematics summarizing that thymol targeting IL4I1 expression to sensitize the PD‐1 blockade in LUAD. Thymol is an effective small compound targeting IL4I1 transcription and inhibiting IL4I1 downstream signaling‐AHR in LUAD cells, which eventually promotes the activation of the immune microenvironment to sensitize the PD‐1 blockade.

Taken together, we reveal that IL4I1 is a key gene modulating the efficacy of PD‐1 blockade in LUAD through blocking the AHR pathway. We further identify thymol as an effective small compound targeting IL4I1 expression through drug screening. Thymol significantly reactivates the immune microenvironment to sensitize the PD‐1 blockade in LUAD via targeting IL4I1‐mediated AHR activation, which provides a novel strategy for the combination immunotherapy for LUAD (Figure 7).

## 3 DISCUSSION

As the most promising antitumor therapy at present, ICBs have changed the landscape for the treatment of cancers. Although ICBs provide unprecedented hope for cancer patients, most patients still fail to respond to ICBs. As NSCLC has a high TMB, the ICBs have well‐expected efficacy and have been widely used in the treatment for NSCLC patients. Many anti‐PD‐1/PD‐L1 antibodies, such as atezolizumab and nivolumab, have been used for the first‐line treatment for advanced NSCLC patients. However, it has been estimated that the response rate of ICBs in NSCLC patients is only about 20%, and the proportion of patients with acquired resistance during treatment is as high as 32%−64%.^2^, ^23^, ^24^ A recent review has pointed out the top 10 challenges in cancer immunotherapy, which is multiagent cancer immunotherapy combination regimens for optimizing long‐term survival.^25^ Therefore, it is necessary to develop novel combination regimens.

Trp/AHR pathway has been proven to drive immunoinhibitory microenvironment in cancers, and is considered to be a hotspot for drug development in cancer combination immunotherapy. We first show that IL4I1 associates more closely with AHR target genes than IDO1 and IDO2, and demonstrate the essential role of IL4I1 in sensitizing the PD‐1 blockade. The IL4I1‐silenced tumors are nearly undetected upon the treatment of anti‐PD‐1 antibody meaning that targeting IL4I1 provides a hopeful avenue for the combination immunotherapy of LUAD. However, there are still no effective drugs targeting IL4I1. Since IL4I1 has been reported to have immunoinhibitory effects and tumor‐promoting effects in several cancer types, the development of IL4I1‐targeted drugs will provide a novel strategy for the treatment of numerous cancer types.

Natural small molecules are highly valuable in the realm of drug development because of their low toxicity, superior distribution capacity, and abundant availability.^26^ The widely used antitumor drugs, that is, paclitaxel and vincristine, are extracted from taxus and periwinkle, respectively. Camptothecin is a natural small molecule extracted from camptotheca acuminata, and its derivatives, topotecan and irinotecan, have been widely used in tumor chemotherapy. Therefore, we aim to develop an effective natural small molecule targeting IL4I1 transcription. Through the screening of a drug library containing 720 natural small molecules, we identify thymol as an effective small molecular targeting IL4I1 transcription.

Thymol, which chemical name is chemicllay‐isopropyl‐5‐methylphenol, is a natural monoterpene phenol often derived from thyme species. Thyme is widely used in traditional medicine in China for its antitussive, expectorant, and acesodyne properties. The pharmacokinetic properties of thymol have been revealed in several previous studies.^27^, ^28^ Thymol exhibits a diverse array of biological and therapeutic activities, including antifungal, antileishmanial, and antiviral properties.^29^, ^30^, ^31^ In recent years, the antitumor effect of thymol has been observed. Several studies have shown that thymol has a cytotoxicity effect on cancer cells, such as HepG2, Caco‐2, K562, Hela cells, and H1299. However, the IC50 is quite high, up to 200−500 μM.^32^ Furthermore, in vitro cancer studies have shown that thymol has the ability to induce apoptosis, generate reactive oxygen species dose‐dependently, and exhibit antioxidant effects, particularly at concentrations greater than 100 μM.^32^, ^33^, ^34^ Therefore, the previous in vitro evidence confirm the antitumor effect of thymol, but the effective concentration is relatively high and the mechanism has not been fully elucidated. The in vivo antitumor evidence of thymol is still limited. Zeng et al. and Hassan et al. found that thymol could inhibit the progression of colorectal cancer.^35^, ^36^ De La Chapa et al. showed that thymol had antitumor effects against oral squamous cell carcinoma.^37^ Our study first reveals the strong effect of thymol on the activation of antitumor immune responses based on the in vivo evidence.

Our study also has several limitations. First, we do not use a humanized mouse model or patient‐derived tumor xenograft mouse model to evaluate the therapeutic effect of thymol. These models could provide a more accurate representation of the human TME and allow for a more comprehensive evaluation of thymol's therapeutic effects. Second, further study needs to explore the direct drug target to optimize the drug structure for higher antitumor activity.

In summary, we show that thymol targeting IL4I1 expression remodels the immune microenvironment to sensitize the PD‐1 blockade in LUAD. The tumors are almost repressed upon the combination treatment of thymol and anti‐PD‐1 antibody, meaning that thymol is a strong sensitizer for the PD‐1 blockade in LUAD. Our study provides a novel strategy for the combination immunotherapy of LUAD.

## 4 MATERIALS AND METHODS

### 4.1 Cell culture

LLC cells were obtained from the Cell Bank of the Chinese Academy of Sciences. A549 and HEK 293T cells were obtained from the American Type Culture Collection. DMEM medium was used for culturing LLC cells and HEK 293T cells, and RPMI‐1640 medium was used for culturing A549 cells.

### 4.2 Analysis of LUAD patient samples

We obtained human LUAD and paired adjacent NTs from LUAD patients who had an operation during 2021−2022 in the Department of Lung Cancer Surgery, Tianjin Medical University General Hospital. The clinical information of the patients is shown in Table S2. The histopathology of patients was confirmed by clinical doctors and pathologists. Primary LUAD samples and paired NTs were dissected into two parts: one part stored in TRIzol reagent (Invitrogen) to extract the RNA and protein, and the other part fixed in 4% paraformaldehyde for IHC staining. In order to examine the association between the efficacy of ICBs and IL4I1 expression, we collected 10 clinical LUAD biopsy specimens before the treatment of PD‐1/PD‐L1 blockade with chemotherapy. Responder refers to the patients with PFS > 12 months. Nonresponder refers to patients with PFS≤ 12 months. The clinical information of the patients treated with anti‐PD‐1/anti‐PD‐L1 antibodies is listed in Table S3. The human patient sample‐based studies were performed in accordance with the ethics guidelines of the committee of Nankai University and Tianjin Medical University General Hospital based on the Declaration of Helsinki (Ethic approved number: IRB2022–KY−060). All LUAD patients from Tianjin Medical University General Hospital provided informed consent.

### 4.3 Bioinformatic analysis

The information about the IL4I1 mRNA level in LUAD and NTs was obtained from the GTEx database and TCGA database. The information about the protein level of IL4I1 in LUAD and NTs was obtained from the CPTAC database. The association between the survival of LUAD patients and IL4I1 expression was analyzed using the Kaplan−Meier plotter available at http://www.kmplot.com/. The coexpression of IL4I1, IDO1, IDO2, and AHR target genes was analyzed using the data from CPTAC (*n* = 110), OncoSG (*n* = 169), and TCGA databases (*n* = 510).

### 4.4 Establishment of stable cell lines

The sequences of shRNAs targeting murine IL4I1 were 5′ AAA AGC AAG AAA GCC ATG AAT AAG TTT GGA TCC AAA CTT ATT CAT GGC TTT CTT GC 3′ (shIL4I1‐1), and 5′ AAA AGC CCA TCG CGC CTC ATA TTC TTT GGA TCC AAA GAA TAT GAG GCG CGA TGG GC 3′ (shIL4I1‐2). LLC cells infected with lentivirus expressing shIL4I1 or shCtrl were selected with 2 μg/mL puromycin (Thermo‐Fisher Scientific).

### 4.5 Cell proliferation and migration assays

For cell proliferation assay, 1000 cells were seeded on 96‐well plates evenly, the cells were treated with 5 μM thymol (HY‐N6810, MedChemExpress) or DMSO and counted every 24 h until they reached nearly 100% confluency. The migration assay was conducted using Transwell Permeable Supports System (Corning Inc.). A total of 3×10^4^ LLC cells or 5×10^4^ A549 cells in medium with 2% FBS were seeded into the upper chamber of an 8‐μm Millipore transwell insert. The lower chamber was filled with medium supplemented with 10% FBS. Following a 6‐h incubation period, the cells that migrated to the lower surface of the transwell membranes were fixed using 4% paraformaldehyde and subsequently stained with 0.1% crystal violet.

### 4.6 Protein extraction and western blot

The TissueLyser (SCIENTZ‐48) was used to homogenize the tissues, and the TRIzol reagent was utilized to extract the total protein. The RIPA lysis buffer was used to prepare whole cell extracts. The NE‐PER Nuclear and Cytoplasmic Extraction Reagents (Thermo‐Fisher Scientific) were utilized to extract the protein from the nucleus and cytoplasmic regions. The concentrations of proteins were measured using the Pierce BCA Protein Assay Kit from Thermo‐Fisher Scientific. The procedure of western blot was described previously.^38^ The primary antibodies used in the western blot are listed in Table S4.

### 4.7 RNA extraction and qRT‐PCR

TRIzol reagent was used to extract the total RNA, which was then converted to cDNA using M‐MLV reverse transcriptase (Promega). The SYBR Green SuperMix from Yeasen Biotech was utilized for qRT‐PCR on the LightCycler96 system by Roche. The program consisted of an initial step at 95°C for 300 s, followed by 45 cycles of a two‐step reaction: 30 s at 95°C and 45 s at 60°C. 2^−ΔΔCt^ method was used to calculate the relative gene expression with β‐actin for the normalization. The primer sequences are listed in Table S5.

### 4.8 H&E, IF, and IHC staining

The procedures of H&E, IF, and IHC staining were described previously.^38^ The primary antibodies used in IF and IHC staining are listed in Table S4.

### 4.9 Orthotopic murine LUAD models

Animal experiments were approved by the Institutional Animal Care and Use Committee of Tianjin Medical University General Hospital (Ethic approved number: IRB2022–DWFL−092). Female C57BL/6 mice (aged between 6and 8 weeks) were purchased from SPF Biotechnology.

1×10^6^ luciferase‐labeled LLC cells were suspended in 50 μL cold phosphate‐buffered saline (PBS) and mixed with 50 μL cold Matrigel (Corning) before being injected into the middle lobe of the right lungs in C57BL/6 mice. To investigate the role of silencing IL4I1 or thymol in enhancing the efficacy of PD‐1 blockade in vivo, mice were randomly divided into four groups at day 5 post‐injection. The mice were intraperitoneally injected with rat IgG2a isotype antibody (BE0089, BioXcell) or 10 mg/kg of mouse anti‐PD‐1 antibody (CD279, BE0146, BioXcell) dissolved in 100 μL PBS every 7 days. The thymol used in the mouse treatment was dissolved in a solvent containing 40% PEG300, 5% Tween‐80, 10% DMSO, and 45% saline. Seventy‐five milligram per kilogram thymol was used to treat mice based on a pilot experiment showing that 50 mg/kg thymol had no significant effect on tumor progression, while 75 mg/kg thymol treatment significantly inhibited tumor progression (data were not shown). The mice were intraperitoneally injected with 75 mg/kg thymol or vehicle control every 2 days. Bioluminescence imaging was used to detect tumor growth.

### 4.10 Flow cytometry

The mice were sacrificed for collecting the tumors. The tumors were then minced with scalpel blades and incubated with the digestion buffer for 30 min at 37°C. Red Blood Cells Lysis Buffer (Solarbio) was utilized to lyse the erythrocytes in tumors. The cells were rinsed with PBS containing 1% FBS and then filtered using a 40‐μm nylon filter. The cells were labeled with specified antibodies for 30 min for cell‐surface marker staining. As for the analysis of macrophages and MDSC, the cells were Fc‐blocked with anti‐CD16/32 before antibody staining. For intracellular staining, surface‐stained cells were fixed and permeabilized with Permeabilization/Wash Buffer (BD Bioscience), and then stained with indicated antibodies. CD45‐PE, CD11b‐APC, F4/80‐APC/CY7, CD206‐FITC, and MHC II‐PERCP/Cy5.5 antibodies were used to detect macrophages. CD45‐PE, CD11b‐APC, Ly6G‐PE/CY7, and Ly6C‐FITC antibodies were used for detecting M‐MDSC and G‐MDSC. CD45‐APC, CD3‐FITC, CD8‐PE/CY7, and GZMB‐PE antibodies were used to detect CD8^+^ T cells. CD45‐APC, CD4‐PE, and CD25‐FITC antibodies were used to detect Tregs. CD45‐PE and NK1.1‐APC antibodies were used to detect NK cells. The primary antibodies used in flow cytometry are listed in Table S4.

### 4.11 ELISA

The concentrations of murine TNF‐α in serum were assayed by murine TNF‐α ELISA Kit (Wuxin Donglin Sci & Tech Development) in accordance with the manufacturer's protocols. The Infinite M200 PRO microplate reader (TECAN) was used to measure the optical density values.

### 4.12 Dual‐luciferase reporter assay

The promoter region (−2760 to −200) of the IL4I1 gene was cloned into the plasmid pGL4.17[*luc2/Neo*] (Promega) to create the pGL4‐pIL4I1 firefly luciferase reporter plasmid. A549 cells were stably transfected with renilla luciferase (RenLuc) control reporter plasmid and pGL4‐pIL4I1 firefly luciferase reporter plasmid. Dual‐Luciferase Reporter Assay System (Promega) was used to measure the luciferase activity.

### 4.13 Statistics

Statistical analysis was performed using Prism 8.0 software (GraphPad Software). Quantitative data were presented as means ± SEM, and the Student's *t*‐test was used to assess the differences between the groups. The continuous variables of the proliferation curve were analyzed using the two‐way ANOVA test. Coexpression of IL4I1 and other genes was evaluated using the Spearman or Pearson test. The differences between the survival curves were determined using the log‐rank test. Statistical significance was defined as *p < 0.05, **p < 0.01, ***p < 0.001, and ns denoted no significance.

## AUTHOR CONTRIBUTIONS

TL and JL designed the study. TL, JS, LW, XQ, and RZ conducted the experiments and acquired data. MD, FR, XL, and YP conducted the bioinformatics analyses. ZZ, YC, and YL conducted the FACS and IHC analyses. TL, SX, and JC acquired consent from patients, acquired clinical samples, and collected clinical data. TL, JL, and YS wrote the manuscript. SX and JC supervised the research and edited the manuscript. All authors have read and approved the final manuscript.

## CONFLICT OF INTEREST STATEMENT

The authors declare that they have no conflict of interest.

## ETHICS STATEMENT

The human patient sample‐based studies were performed in accordance with the ethics guidelines of the committee of Tianjin Medical University General Hospital and Nankai University based on the Declaration of Helsinki (Ethic approved number: IRB2022–KY−060). All LUAD patients from Tianjin Medical University General Hospital provided written informed consent. Animal experiments were approved by the Institutional Animal Care and Use Committee of Tianjin Medical University General Hospital (Ethic approved number: IRB2022–DWFL−092).

## Supporting information

Supporting Information

## Data Availability

The datasets used and/or analyzed during the present study are available from the corresponding author on reasonable request.
