# Supplementary material for: Thymol targeting interleukin 4 induced 1 expression reshapes the immune microenvironment to sensitize the immunotherapy in lung adenocarcinoma
Source: MedComm (2020). 2023 Aug 30;4(5):e355. doi: 10.1002/mco2.355 (PMC10466095; doi:10.1002/mco2.355)
Supplement: Supplementary file 1 — Supporting Information [file MCO2-4-e355-s001.pdf]

Thymol targeting IL4I1 expression reshapes the immune microenvironment to sensitize the immunotherapy in lung adenocarcinoma

Running Title: Thymol targeting IL4I1 sensitizes immunotherapy

Tong Li[1,3#], Jie Shi[2#], Longlong Wang[2#], Xuan Qin[4], Rui Zhou[2], Ming Dong[1,3], Fan Ren[1,3], Xin Li[1,3], Zihe Zhang[1,3], Yanan Chen[2], Yanhua Liu[2], Yongjun Piao[2], Yi Shi[2], Song Xu[1,3*], Jun Chen[1,3*], Jia Li[2*]

[1]Department of Lung Cancer Surgery, Tianjin Medical University General Hospital, Tianjin, 300052, China.

[2]School of Medicine, Nankai University, Tianjin 300071, China.

[3]Tianjin Key Laboratory of Lung Cancer Metastasis and Tumor Microenvironment, Lung Cancer Institute, Tianjin Medical University General Hospital, Tianjin, 300071, China.

[4]Department of Thyroid and Neck Tumor, Tianjin Medical University Cancer Institute and Hospital, Tianjin, 300060, China.

[#]Tong Li, Jie Shi and Longlong Wang contributed equally to this work.

Corresponding author

Jia Li, E-mail: lijia0731@nankai.edu.cn, School of Medicine, Nankai University, 94 Weijin Road, Tianjin 300071, China; Phone: (86)-22-23509482, Fax: (86)-22-23502554.; Jun Chen, E-mail: hunterchenjun@hotmail.com; Song Xu, E-mail: xusong198@hotmail.com.

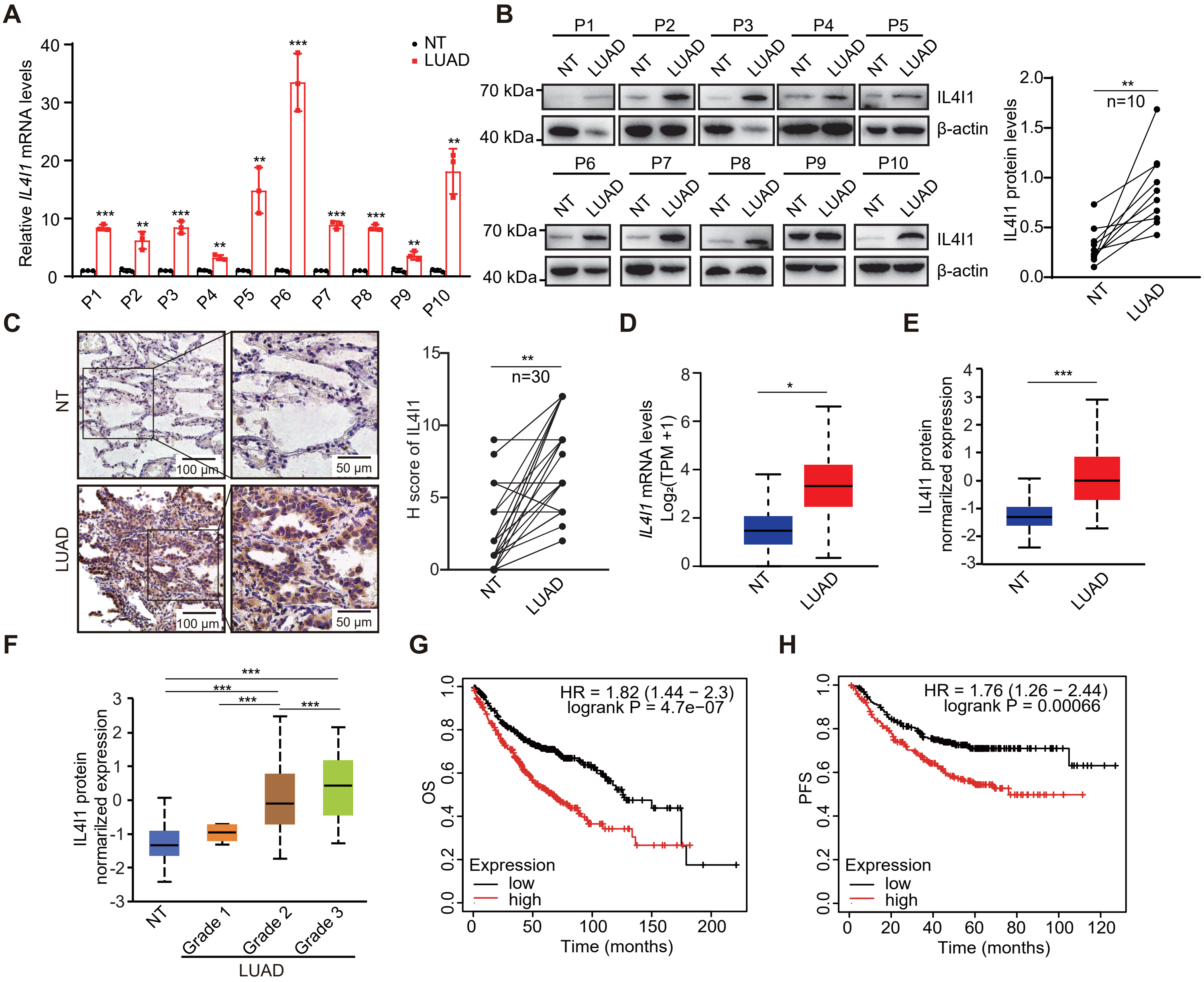

Figure S1. IL4I1 is high expressed in tumors and negatively correlated with the outcomes of LUAD patients.
A. qRT-PCR analysis of *IL4I1* in LUAD tumors and paired adjacent normal lung tissues (NT) (n=10). **B.** Western blot analysis of IL4I1 in LUAD tumors and paired adjacent normal lung tissues (NT), and the quantification results of relative IL4I1 protein levels (n=10). **C.** Representative images of IHC analyses of IL4I1 in LUAD tumors and paired adjacent normal lung tissues (NT), and quantification by H-score (n = 30). **D.** The mRNA levels of *IL4I1* in LUAD tumors (n=483) and normal lung tissues (NT, n=347) from GTEx database and TCGA database. **E.** The protein levels of IL4I1 in LUAD tumors (n=111) and paired normal lung tissues (NT, n=111) from CPTAC database. **F.** The protein levels of IL4I1 in normal lung tissues (NT, n=111) and LUAD tumors with different grades (Grade 1, n=7; Grade 2, n=59; Grade 3, n=39). **G-H.** Kaplan-Meier survival plot to show the overall survival (OS) (G) and progression free survival (PFS) (H) of LUAD patients with different IL4I1 expression. Quantification data are plotted as means ± SEM from three independent measurements. *P < 0.05, **P < 0.01, ***P < 0.001, by two-sided student's t-test.

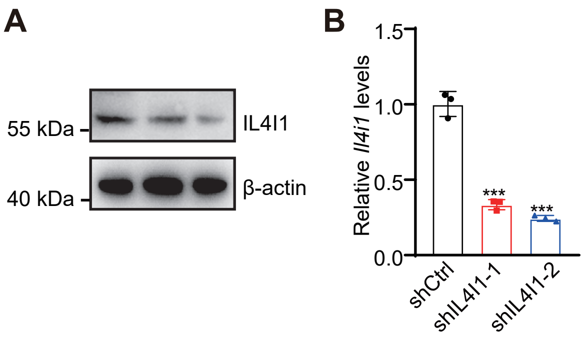

Figure S2. The construction of IL4I1-silenced LLC cells.
A-B. Western blot (A) and qRT-PCR (B) analyses of IL4I1 in control shRNA (shCtrl)- or IL4I1 shRNA (shIL4I1-1/IL4I1-2)-transfected LLC cells.Quantification data are plotted as means ± SEM from three independent measurements. ***P < 0.001, by unpaired two-sided student’s t-test.

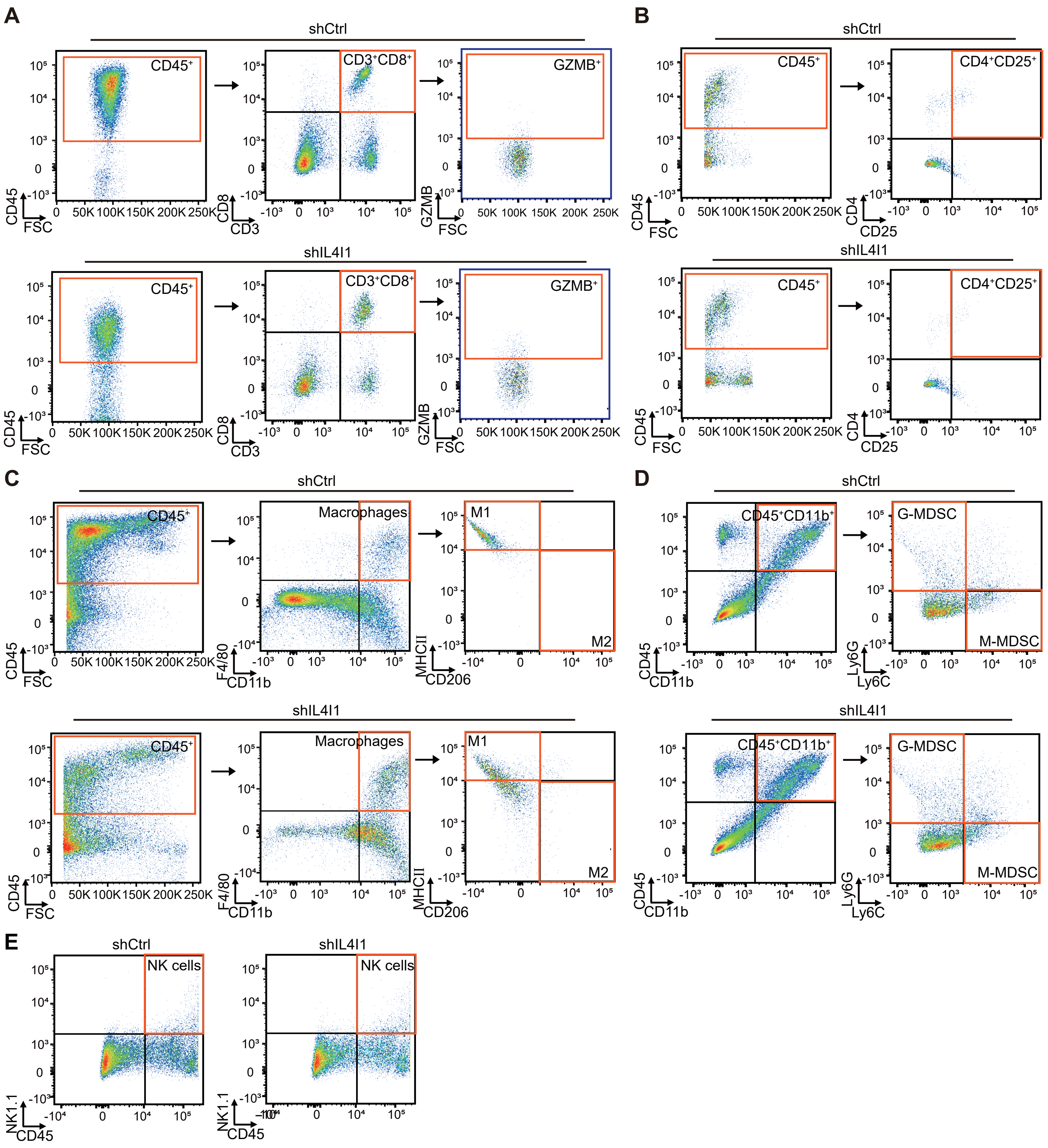

Figure S3. The flow cytometry analyses of tumor infiltrated-immune cells in mice orthotopically inoculated with shCtrl and shIL4I1 LLC cells. A-E. The representative scatter plots and gating strategies of $CD8^+$ T cells (A), granzyme B^+ ($GZMB^+$) T cells (A), regulatory T cells (Tregs, B), macrophages (C), myeloid derived suppressor cells (MDSCs, D) and natural killer (NK) cells (E) in LUAD tumors formed in C57BL/6 mice.

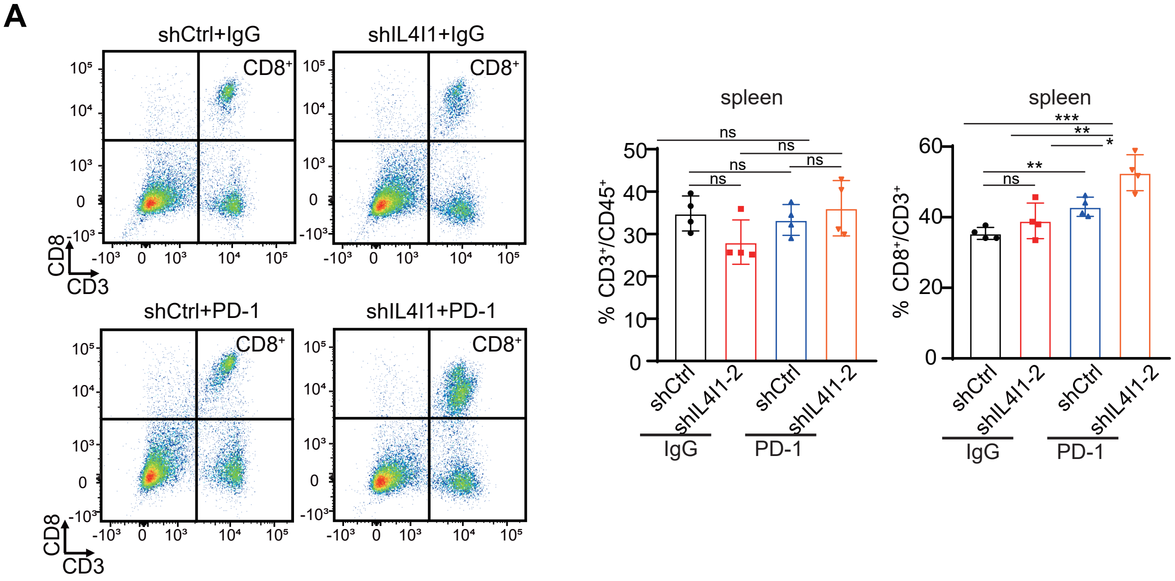

Figure S4. The proportion of CD8⁺ T cells in spleen of the mice orthotopically inoculated with shCtrl and shIL4I1 LLC cells with the treatment of anti-PD-1 antibody.

A. The representative scatter plots and statistical analyses of CD3⁺ T cells and CD8⁺ T cells in the spleen of the mice. Quantification data are plotted as means ± SEM. ns not significant, *P < 0.05, **P < 0.01, ***P < 0.001, by unparied two-sided student's t-test.

A

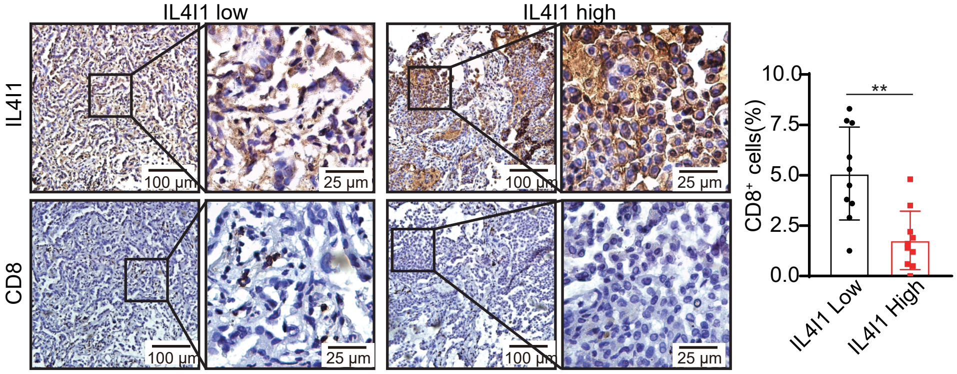

Figure S5. CD8⁺ T cells infiltration in tumors with different IL4I1 expression in LUAD patients.
A. Representative images of IHC analysis of CD8⁺ T cells infiltration in tumors with different IL4I1 expression in LUAD patients with the quantification analyses. Quantification data are plotted as means ± SEM. **P < 0.01 by unparied two-sided student's t-test.

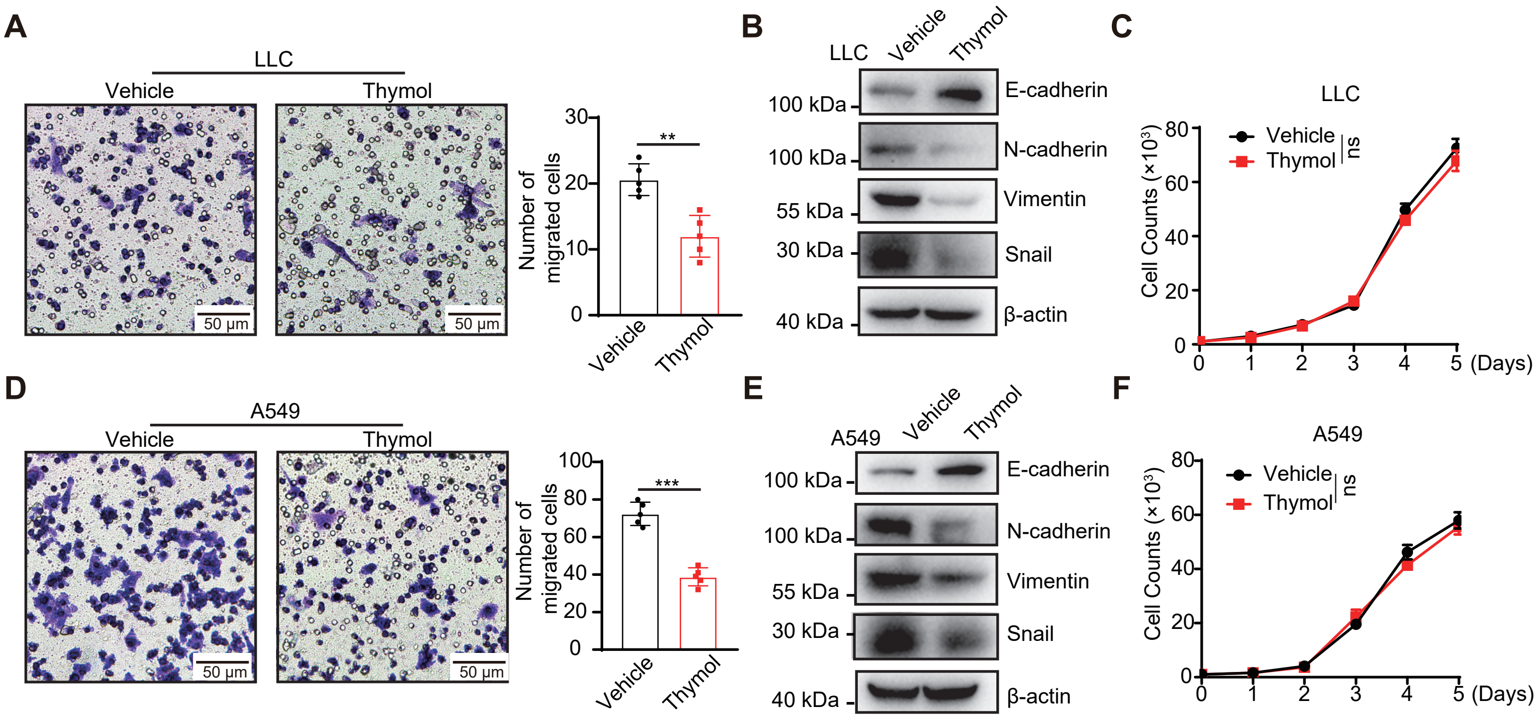

Figure S6. Thymol inhibits the epithelial-mesenchymal transition (EMT) process and the motility ability of LUAD cells.
A. Transwell cell migration assay of LLC cells treated with 5 μM thymol for 48 h. **B.** Western blot analysis of EMT markers in LLC cells treated with 5 μM thymol for 48 h. **C.** The proliferation curves of LLC cells treated with 5 μM thymol. **D.** Transwell cell migration assay of A549 cells treated with 20 μM thymol for 48 h. **E.** Western blot analysis of EMT markers in A549 cells treated with 20 μM thymol for 48 h. **F.** The proliferation curves of A549 cells treated with 20 μM thymol. Quantification data are plotted as means ± SEM from three independent measurements. ns not significant, $^{**}P < 0.01$, $^{***}P < 0.001$, by unpaired two-sided student's t-test for A and D, by two-way ANOVA test for C and F.

A

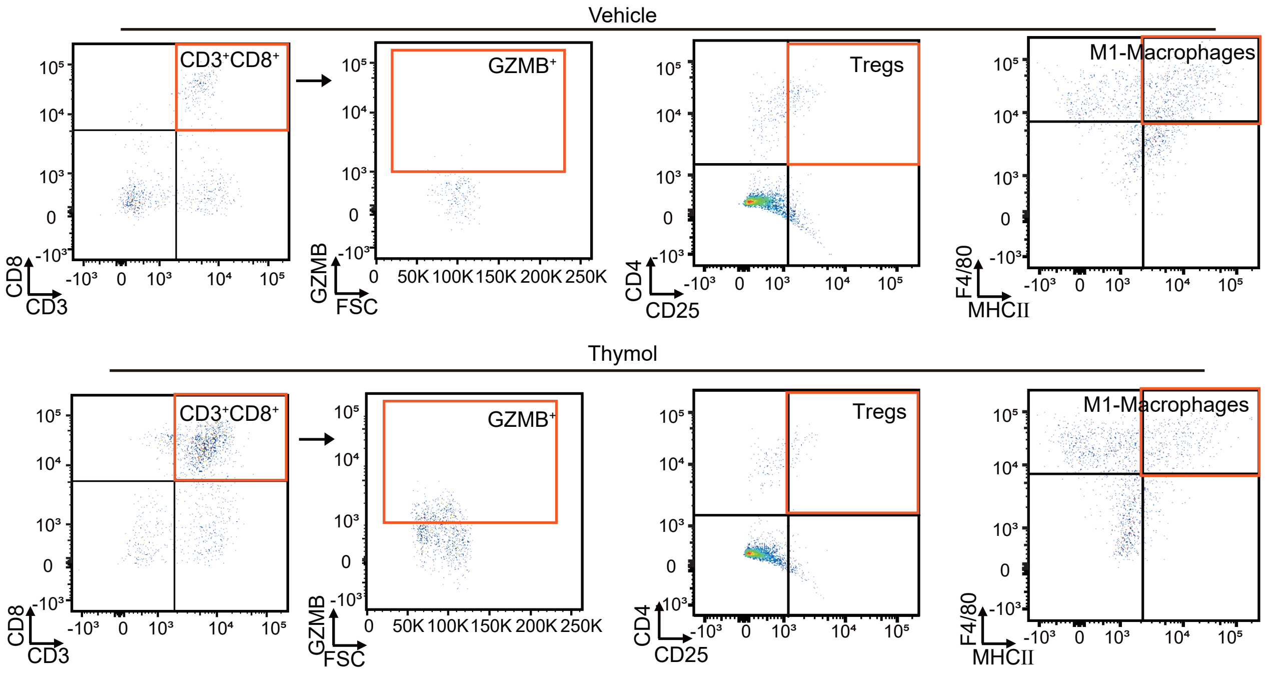

Figure S7. The flow cytometry analyses of tumor infiltrated-immune cells in LUAD tumors treated with vehicle or thymol.
A. The representative scatter plots of CD8⁺ T cells, granzyme B⁺ (GZMB⁺) T cells, regulatory T cells (Tregs) and macrophages in LUAD tumors formed in C57BL/6 mice treated with vehicle or thymol.

Table S1. Effects of thymol on blood biochemical indexes in mice

	Vehicle	Thymol	Reference value
TP (g/L)	50.73±0.24	52.53±1.71	36-66
ALB (g/L)	34.97±0.92	32.87±1.65	25-48
GLO (g/L)	15.77±0.76	19.67±0.29	5-40
ALT (U/L)	57.00±10.61	55.00±2.16	28-132
AST (U/L)	131.33±11.47	186.33±20.81	59-247
ALP (U/L)	215.00±43.86	202.33±33.07	62-209
CRE (umol/L)	44.33±12.36	71.00±8.60	26-88
BUN (mmol/L)	11.53±1.44	9.09±0.33	4.9-10.4
TBIL (umol/L)	3.88±0.84	3.76±0.64	1.7-15.4
TBA (umol/L)	5.82±1.18	6.53±1.55	0-15
TG (mmol/L)	1.39±0.37	1.28±0.37	0.62-1.63
CHOL (mmol/L)	3.07±0.42	2.54±0.04	0.93-2.48
GLU (mmol/L)	11.29±1.88	8.93±0.91	3.49-10.67
Ca (mmol/L)	2.09±0.07	2.16±0.00	0.82-2.18
P (mmol/L)	2.93±0.46	2.81±0.10	0.75-3.17
Mg (mmol/L)	1.29±0.06	1.09±0.01	0.33-1.59

All values are shown as the means ± SD (n = 3 in each group). TP, total protein; ALB, albumin; GLO, globulin; ALT, alanine aminotransferase; AST, aspartate aminotransferase; ALP, alkaline phosphatase; CRE, creatinine; BUN, blood urea nitrogen; TBIL, total bilirubin; TBA, total bile acid; TG, triglyceride; CHOL, cholesterol; GLU, glucose.

Table S2. Characteristic of patients

	N (%)
Gender	
Male	19 (63.33%)
Female	11 (36.67)
Age	
＜60	8 (26.67%)
≥60	22 (73.33%)
Smoker	14 (46.67%)
Histology	
Adenocarcinoma	30 (100%)
Pathological Stage	
Ⅰ	14 (46.67%)
Ⅱ	12 (40%)
Ⅲ	3 (10%)
Ⅳ	1 (3.33)

Table S3. Characteristic of patients treated with anti-PD-1/anti-PD-L1 antibody

	Age (years)	Gender	Histology	Stage	Treatment	PFS (months)	Responder
P1	59	Female	Adenocarcinoma	IIIb	AP*+ Pembrolizumab	13	Yes
P2	71	Female	Adenocarcinoma	IIIb	AP+ Pembrolizumab	4	No
P3	49	Male	Adenocarcinoma	IIIc	AP+ Atezolizumab	11	No
P4	56	Female	Adenocarcinoma	IVb	AP+ Pembrolizumab	19	Yes
P5	67	Male	Adenocarcinoma	IIIc	AP+ Atezolizumab	8	No
P6	66	Male	Adenocarcinoma	IVa	AP+ Atezolizumab	23	Yes
P7	58	Female	Adenocarcinoma	IVa	AP+ Pembrolizumab	14	Yes
P8	74	Male	Adenocarcinoma	IIIb	AP+ Pembrolizumab	5	No
P9	61	Male	Adenocarcinoma	IVa	AP+ Pembrolizumab	28	Yes
P10	53	Female	Adenocarcinoma	IVa	AP+ Pembrolizumab	17	Yes

*AP: Pemetrexed + Cisplatinum

Table S4. List of antibodies

Antibody Name	Company	Cat No.	Usage	Dilution
IL4I1	Invitrogen	PA5-113266	WB, IHC,	1:1000
β-actin	Immunoway	YM3028	WB	1:5000
E-Cadherin	BD Biosciences	610182	WB	1:1000
Vimentin	Cell Signaling Technology	5741S	WB	1:1000
N-Cadherin	Proteintech	22018-1-AP	WB	1:1000
Snail	Cell Signaling Technology	3879	WB	1:1000
AHR	Proteintech	67785-1-Ig	WB, IF	1:1000
α-Tubulin	Proteintech	66031-1-Ig	WB	1:1000
Lamin A/C	Proteintech	10298-1-AP	WB	1:1000
CD8	Proteintech	66868-1-Ig	IHC	1:5000
GZMB	Bioss	bs1351R	IHC	1:500
CD16/CD32	eBioscience	14-0161-82	FACS	1:200
CD45-PE	Biolegend	103106	FACS	1:200
CD11b-APC	Biolegend	101212	FACS	1:200
F4/80-APC/CY7	Biolegend	123118	FACS	1:200
MHCⅡ-PERCP/CY5.5	Biolegend	141704	FACS	1:200
CD206-FITC	Biolegend	141704	FACS	1:200
Ly6G-PE/CY7	Biolegend	127618	FACS	1:200
LY6C-FITC	Biolegend	128006	FACS	1:200
CD45-APC	Biolegend	103106	FACS	1:200
CD3-FITC	Biolegend	100204	FACS	1:200
CD8-PE/CY7	eBioscience	25-0081-82	FACS	1:200
GZMB-PE	ebioscience	12-8898-80	FACS	1:200
CD4-PE	ebioscience	12-0042-82	FACS	1:200
CD25-FITC	Biolegend	101908	FACS	1:200
NK1.1-APC	Biolegend	108710	FACS	1:200

Table S5. Primes for qPCR

Primer	Forward Sequence (5'- 3')	Reverse Sequence (5'- 3')
Mouse		
IL4I1	TGCCCAAGAGAGCTGAAGACAG	ACTACCACCTTCTGGGGCTT
β-actin	GGCTGTATTCCCCTCCATCG	GCACAGGGTGCTCCTCAG
IL6	AGTCCTTCCTACCCCAATTTCCA	AACGCACTAGGTTTGCCGAG
IL10	GGGTTGCCAAGCCTTATCGG	GTAGACACCTTGGTCTTGGAGC
IL22	GTGCGATCTCTGATGGCTGT	AGTTTCTCCCCGATGAGCCG
IL1B	TGCCACCTTTTGACAGTGATGA	TGCCTGCCTGAAGCTCTTGT
TIPARP	AAAGGTTGCGTCCTGTGCTT	AGTGGCACCGTTTCCAAGTT
MMP13	TGGTCTTCTGGCACACGCTT	AGGGTTGGGGTCTTCATCGC
CYP1B1	AGCCAAGTGGCCTAACCCAG	TGCACTGATGAGCGAGGATGG
Human		
IL4I1	TTGCGCAGCTTCACTTCGTG	GTGATTGTGGTTGGCGCTGG
β-actin	CGTCACCAACTGGGACGA	ATGGGGGAGGGCATACC
IL6	GCAAGGGTCTGGTTTCAGCC	TCGCTCCCTCTCCCTGTAAGT
IL10	CATCAGGGGCTTGCTCTTGC	GTTAGGCAGGTTGCCTGGGA
IL22	TCCTTCCCCAGTCACCAGTTG	GCTAGCCTCCTTAGCCAGCAT
IL1b	CTCGCCAGTGAAATGATGGCT	GTCGGAGATTCGTAGCTGGAT
TIPARP	GAGGCGCCGAAGGATTTAGT	AAGACGAAGACTTGTGCGCT
MMP1	GCTAACCTTTGATGCTATAACTA	TTTGTGCGCATGTAGAATCTG
CYP1B1	GACGCCTTTATCCTCTCTGCG	ACGACCTGATCCAATTCTGCC
